# Effects of high-dose recombinant human growth hormone treatment on IGF-1 and IGFBP-3 levels in idiopathic dwarfism patients

**DOI:** 10.12669/pjms.38.4.5502

**Published:** 2022

**Authors:** Bin Wu, Honghua Lin, Jian Gao, Juan Sun, Meng Zhao

**Affiliations:** 1 Bin Wu, Department of Emergency Room of Internal Medicine, Anhui Provincial Children’s Hospital, Hefei 230001, Anhui Province, P.R. China; 2 Honghua Lin, Dept. of Endocrinology, Anhui Provincial Children’s Hospital, Hefei 230001, Anhui Province, P.R. China; 3 Jian Gao, Dept. of Endocrinology, Anhui Provincial Children’s Hospital, Hefei 230001, Anhui Province, P.R. China; 4 Juan Sun, Dept. of Endocrinology, Affiliated Hospital of Xuzhou Medical University, 99 Huaihai West Road, Xuzhou 221002, Jiangsu Province, P.R. China; 5 Meng Zhao, Department of Endocrinology, Affiliated Hospital of Xuzhou Medical University, 99 Huaihai West Road, Xuzhou 221002, Jiangsu Province, P.R. China

**Keywords:** Idiopathic dwarfism, Recombinant human growth hormone, Different doses, Curative effect, Growth and development

## Abstract

**Objective:**

To investigate the effects of high-dose recombinant human growth hormone(rhGH) treatment on insulin-like growth factor-1 (IGF-1) and insulin-like growth factor binding protein-3 (IGFBP-3) levels in patients with idiopathic dwarfism.

**Methods:**

This study retrospectively investigated records of idiopathic dwarfism patients treated at Anhui Children’s Hospital or the Affiliated Hospital of Xuzhou Medical University between May 2019 and October 2020. The study identified 76 patients, which were divided based on rhGH treatment dosage into high- and low-dose groups.

**Results:**

The high-dose group showed a total efficacy of 95.3%, which was significantly higher than the 79.41% observed in the low-dose group (P<0.05). Moreover, height, weight, IGF-1 levels, and IGFBP-3 levels were all significantly elevated in the high-dose group compared to the low-dose group (P<0.05). No significant difference in adverse reaction incidence was observed between high- (9.5%) and low-dose (8.8%) groups (P>0.05).

**Conclusions:**

Higher doses of rhGH can provide improved curative effects in patients with idiopathic short stature, likely via elevated levels of IGF-1 and IGFBP-3.

## INTRODUCTION

Idiopathic dwarfism is defined as height that is below the 3^rd^ percentile or is two standard deviations below the median for healthy age, gender, and race-matched children.^1^ Idiopathic dwarfism seriously affects pediatric physical and mental well-being and is accompanied by social and psychological problems ranging from decreased learning to elevated suicide risk. Currently, pathogenic causes for idiopathic dwarfism are not clear, and no consensus treatment practice has been established.^2^ Recent research has identified the human growth factor (HGF)-insulin-like growth factor (IGF) neuroendocrine axis as a pathway of interest. This signaling axis can affect growth and development through abnormal modulation of HGF levels.^3^ As such, current clinical treatment for idiopathic dwarfism largely centers around the restoration of human growth hormone levels, mainly through exogenous administration. Presently, the recommended dose for pediatric patients in China is 0.1-0.2 IU/(kg·d). Initially, lower dosages around 0.1 IU/(kg·d) were preferred for safety reasons, but newer studies indicate that dosing around 0.2 IU/(kg·d) can provide more clinical benefit without a concurrent increase in adverse reactions.^4^

Our objective was to analyze and compare the clinical effects of different doses of rhGH in the treatment of idiopathic dwarfism and its effects on the levels of IGF-1 and IGFBP-3.

## METHODS

This study retrospectively examined records of 76 pediatric patients with idiopathic dwarfism treated at Anhui Children’s Hospital (46 cases) or the Affiliated Hospital of Xuzhou Medical University (30 cases) between May 2019 and October 2020.

### Inclusion criteria:

- Diagnosis of idiopathic dwarfism (The height two standard deviations lower than the average height of children of the same age and sex);^5^
- The bone age of 7~12 years for male and 6~10 years for female patients;
- The growth rate of <4cm per year, the bone age more than two years lower than the actual age;
- Normal intellectual development;
- Complete medical records of pathology and treatment, and no relevant treatment in the three months prior to admission.

### Exclusion criteria:

- Congenital malformations, chromosome abnormalities;
- Metabolic and immune systemic diseases;
- Thyroid diseases, such as hyperthyroidism and hypothyroidism;
- Severe kidney and heart organ diseases;) Mental/psychological diseases.

This study was approved by the medical ethics committee of Anhui Children’s Hospital (Approval number: LC20210805014, Date: 2021-04-30). Guardians provided informed consent for all pediatric patients.

Patients were treated either with low- or high-dose (rhGH) regimens [according to the relevant rhGH diagnosis and treatment consensus of the Chinese Medical Association and the American Society of Clinical Endocrinology, the dosage is 0.1 ~ 0.2U/(kg·d)].^6^,^7^ Post-admission, children were also treated with vitamins, as well as supplements for trace elements and calcium. Dietary consultation was performed. rhGH (Changchun Jinshai Pharmaceutical Co., Ltd., S10980101) was administered intramuscularly around the umbilicus and the middle, front, and outer sides of the thigh prior to bed as a 12-month course. According to the treatment records, 34 patients received treatment with a dose of 0.1 IU/(kg·d) and were set as the low-dose group; 42 patients received a dose of 0.2 IU/(kg·d) and were set as the high-dose group.^8^

Patients were followed up for 12 months to obtain information on height and weight changes, insulin-like growth factor-1 (IGF-1) and insulin-like growth factor binding protein-3 (IGFBP-3) levels before treatment and after 12 months of treatment [(by analyzing 3 ml fasting venous blood samples collected on the day of admission and day of course completion (UniCel DxI800)], adverse events such as lower limb edema, elevated blood glucose, and hypothyroidism, and clinical efficacy-defined as the amount of total height growth.^9^,^10^ An annual growth rate >10cm was considered significant, a rate between 6-10 cm was considered effective, and anything less was considered invalid. The total treatment efficacy was calculated as the combined number of significant and effective clinical efficacies, divided by the total number of patients.

Data was processed using SPSS v.22.0 software. Counting data was presented as n (%) and compared using chi-squared tests. Measurement data was presented as mean±standard deviation. Data with normal distributions were examined using t-tests, while data with non-normal distributions were subjected to rank sum test, with *a*=0.05.

## RESULTS

Of the 76 patients included in the study, 42 were male and 34 were female. Patient average age and height did not differ significantly between low- and high-dose groups (P>0.05. Table-I). After the end of the 12-month treatment course, average height and weight increased in both groups. However, the high-dose group showed significantly greater increases (P<0.05. Table-II). The total efficacy in the high-risk group was 95.3%, which was significantly higher than the 79.41% observed in the low-dose group (P<0.05, Table-III).

**Table-I:** General patient information.

Group	n	Center (Anhui/Xuzhou)	Sex (male/female)	Age (years)	Height (cm)
Low dose	34	21/13	21/13	8.67±1.75	112.50±7.20
High dose	42	25/17	24/18	8.83±1.59	112.14±6.78
x^2^/t	-	0.039	1.660	0.408	0.052
P	-	0.842	0.684	0.684	0.958

**Table-II:** Pre- and post-treatment patient height and weight(x¯±s).

Group (n)	Height (cm)		t	P	Weight (kg)		t	P
	
	Admission day	Treatment completion			Admission day	Treatment completion		
Low dose (34)	112.50±7.20	120.35±8.90	17.969	<0.001	22.44±2.61	24.61±3.01	14.598	<0.001
High dose (42)	112.14±6.78	125.28±9.28	21.641	<0.001	22.54±2.99	26.21±3.34	24.261	<0.001
*t*	0.052	2.345	-	-	0.163	2.164	-	-
*P*	0.958	0.022	-	-	0.871	0.034	-	-

**Table-III:** Clinical efficacy [n (%)].

Group	n	Curative effect			Total effective

		Significant	Efficient	Invalid	
Low dose	34	9(26.47)	18(52.94)	7(20.59)	27 (79.41)
High dose	42	31(73.81)	9(21.42)	2(4.7)	40 (95.3)
*x* ^2^	-	16.889	8.146	4.508	17.227
P	-	<0.001	0.004	0.034	<0.001

No significant differences in IGF-1 and IGFBP-3 levels between the two groups were observed prior to the start of the treatment (P>0.05). However, after the treatment, IGF-1 and IGFBP-3 levels in both groups increased, with a higher increase observed in the high-dose group (P<0.05. Table-IV). No significant differences in adverse reaction incidence and severity were observed between the low- and high-dose groups (X^2^=0.011, P=0.916, P>0.05). The high-dose group included two patients with the lower limb edema, and a single case each of hypothyroidism and elevated blood glucose, for a total incidence rate of 4/42 (9.5%). The low-dose group presented one case each of lower limb edema, elevated blood glucose, and hypothyroidism, for a total incidence rate of 3/34 (8.8%).

**Table-IV:** Pre- and post-treatment IGF-1 and IGFBP-3 levels(x¯±sμg/L).

Group (n)	IGF-1		t	P	IGFBP-3		t	P
	
	Admission day	Treatment completion			Admission day	Treatment completion		
Low dose (n=34)	146.79±18.53	275.44±22.82	165.279	<0.001	3.21±0.36	4.25±0.49	40.977	<0.001
High dose (n=42)	147.14±19.31	325.07±23.38	226.915	<0.001	3.19±0.34	5.44±0.57	60.362	<0.001
t	0.080	9.299	-	-	0.176	10.414	-	-
P	0.937	<0.001	-	-	0.861	<0.001	-	-

## DISCUSSION

This study found that the clinical efficacy and insulin-like growth factor level of the high-dose group were significantly better than those of the low-dose group. Di Qie et al. divided 37 children with short stature (less than gestational age) into low-dose rhGH group (0.1 ~ 0.15IU/kg/d) and high-dose rhGH group (0.16~0.2IU/kg/d). After 12 months of treatment, the height increase rate and IGF level in the high-dose group were significantly better than those in the low-dose group.^11^ However, R Coelho et al. conducted a randomized controlled trial of 49 GHD children receiving different doses of r-hGH in adolescence. The results showed that high doses of r-hGH did not seem to have a significant effect on the final height of growth hormone-deficient children. Therefore, rhGH treatment should be carried out before puberty and before epiphyseal closure.^12^ Idiopathic dwarfism accounts for 60-80% of short stature pediatric developmental disorders. Its deleterious impact on pediatric development has considerable psychological ramifications^13^ Furthermore, idiopathic dwarfism is linked to the immature development of physiological systems, potentially increasing the risk of digestive, respiratory, cardiovascular, and cerebrovascular system complications.^14^

Historically, idiopathic dwarfism has been treated with androgens, gonadotropin releasing hormone analogues, and aromatase inhibitors. While these agents can promote physical development to a certain extent, they may have non-ideal long-term effects.^15^ More recently, studies have highlighted the importance of the growth hormone-insulin-like growth factor axis.^16^ Trials involving exogenous administration of recombinant growth hormone showed that this therapy results in increased height in pediatric patients.^17^,^18^ However, the ideal dosage of recombinant growth hormone is unclear, with some guidelines recommending 0.15-0.2 IU/(kg·d) and others proposing 0.1-0.2 IU/(kg·d).^19^ Here, we looked at patients with idiopathic dwarfism treated with low- and high-dose regimens of rhGH. We found that high-dose treatment resulted in greater heights, weights, and overall effectiveness rates. Our study indicates that dosages closer to 0.2 IU/(kg·d) can provide improved curative effects without additional incidences of adverse events.

Growth hormone can also promote growth and development by forming a ternary complex with IGF-1 and IGFBP-3.^20^ Indeed, a study on 50 patients with growth hormone deficiency showed that IGF-I and IGFBP-3 were highly sensitive and specific for diagnosing GHD.^21^ In our study, IGF-1 and IGFBP-3 levels were higher in the high-dose group, indicating that exogenous rhGH supplementation can promote physical development through elevating these two hormones.^22^

### Limitations of the study:

The sample size was relatively small with only few observation indicators that were only investigated at a single time point (12 months) following the start of the treatment. Follow-up investigations will need to incorporate larger sample sizes, involve multiple study centers, and investigate more observational indicators at more time points. This will aid in more comprehensively analyzing the effect of high-dose rhGH treatment in patients with idiopathic dwarfism, as well as its impact on IGF-1 and IGFBP-3 levels.

## CONCLUSIONS

High-dose rhGH used to treat idiopathic dwarfism can yield improved curative effects, promote better growth and development, and boost IGF-1 and IGFBP-3 levels without increasing adverse reactions.

### Authors’ contributions:

**BW & MZ:** Conceived and designed the study.

**HL, JG, and JS:** Collected the data and performed the analysis.

**BW& MZ:** Were involved in the writing of the manuscript and is responsible for integrity of the study. All authors have read and approved the final manuscript.
